# Large-scale global retrospective study on the interaction between ancestry and risk of comorbid autoimmune diseases in patients with pemphigus

**DOI:** 10.1038/s41598-024-78031-z

**Published:** 2024-12-03

**Authors:** Rochi Saurabh, Anikamila Cani, Marius Möller, Hauke Busch

**Affiliations:** Lübecker Institute for Experimental Dermatology (LIED), Lübeck, Germany

**Keywords:** Epidemiology, Skin diseases, Autoimmune diseases, Autoimmunity

## Abstract

The pemphigus family of skin blistering diseases represents a rare yet potentially life-threatening condition characterized by multiple known genetic loci associated with other autoimmune disorders. While several studies have empirically indicated an increased risk of developing additional autoimmune diseases in individuals with pemphigus, the scarcity of data and the rarity of pemphigus have hindered efforts to establish and generalize these associations across diverse populations. In this study, we analyzed a dataset comprising 126 million patients, including 18,000 with pemphigus, to assess the likelihood of developing any of 74 autoimmune diseases following a diagnosis of pemphigus. For a subset of 26 diseases from this list with adequate patient numbers, we conducted further case-control retrospective analyses to quantify the odds and hazard ratios of developing comorbid conditions across various ethnicities. Our findings reveal highly significant and generalizable associations between pemphigus and pemphigoid diseases, discoid lupus erythematosus, lichen planus, and undifferentiated connective tissue disease, among others.

## Introduction

Pemphigus comprises a group of rare autoimmune skin blistering diseases associated with a significantly elevated mortality risk^1,2^. These diseases are characterized by the production of auto-antibodies targeting proteins within desmosomes, leading to disrupted adhesion and blister formation^3^. Notably, pemphigus vulgaris (PV), the most prevalent variant, is linked to anti-desmoglein 3 (DSG3) auto-antibodies, while pemphigus foliaceus (PF) and erythematosus (PE) are associated with auto-DSG-1 auto-antibodies^4^. At the genetic level, pemphigus is linked to variants in the HLA class II genes, the HSP70 family, MHC2TA variants, and several other loci, many of which are also implicated in other autoimmune diseases^5,6^. Due to their rarity, comprehensively understanding all subtypes of pemphigus has proven challenging, particularly regarding the scale needed to investigate associations with other rare autoimmune diseases.

Evidence suggests a tendency for autoimmune diseases to co-occur, referred to as “multiple autoimmune syndrome (MAS)”^7^ or “polyautoimmunity”^8^. Several mechanisms have been proposed to explain the increased risk of MAS, including genetic predisposition, environmental triggers such as the gut microbiome and obesity, and complex mediators^9–14^.

A nationwide case-control study conducted in Taiwan^15^ identified an elevated risk of developing Sjögren’s syndrome, systemic lupus erythematosus, and psoriasis. Smaller studies conducted in Israel and the US^16,17^ also reported associations between autoimmune thyroid diseases and rheumatoid arthritis. A review of pemphigus epidemiology^18^ corroborated these findings and highlighted a novel association with psoriasis^19,20^ and ulcerative colitis^21^. Limited evidence exists for a link between myasthenia gravis and paraneoplastic pemphigus^22,23^. However, the generalizability of these associations across different ethnic groups remains untested.

In this study, we aim to investigate several autoimmune diseases suspected of being clinically associated with pemphigus and examine their interaction with patient ancestry. Leveraging TriNetX^24^, a global health research network providing access to anonymized longitudinal electronic medical records across healthcare organizations (HCOs), allows us to assess autoimmune comorbidities of pemphigus at a scale not feasible with other resources. This retrospective epidemiological cohort study encompasses all 126 million individuals in the database. Of these, approximately 18,000 are classified under ICD10CM-L10, representing general pemphigus diagnoses, predominantly comprising pemphigus vulgaris and pemphigus foliaceus. For each individual in the pemphigus group, we assign a non-pemphigus match based on age, age at onset and gender. Additionally, we examine ethnicity-specific cohorts matched for propensity.

As a result, we quantify the overall likelihood of developing a comorbidity after pemphigus for 74 separate autoimmmune diseases and find that the pemphigoid diseases, psoriasis, rheumathoid arthritis and Sjögren syndrome are the most likely to develop. Of these, 26 fulfill a minimum requirement of a size 30 case count to test for significance compared to a control cohort. 15 diseases are significantly comorbid in the largest (white) testing group while 9 are non-significantly comorbid. The most consistently significantly comorbid diseases accross ethnicities are the pemphigoid diseases, discoid lupus erythematosus, lichen planus, and undifferentiated connective tissue disease.

## Methods

The TriNetX database was utilized to conduct both preliminary and follow-up analyses aimed at identifying risk factors associated with concomitant autoimmune illnesses in pemphigus patients. Data from 108 healthcare organizations (HCOs) underwent analysis on the TriNetX platform from October 29th to November 12th, 2023. To ensure compliance with privacy regulations regarding health information, TriNetX withheld specific details regarding the participating HCOs, thereby preventing the disclosure of geographical or institutional information.

TriNetX is compliant with the Health Insurance Portability and Accountability Act (HIPAA), the US federal law which protects the privacy and security of healthcare data^25^.

### Study design

**Fig. 1:**
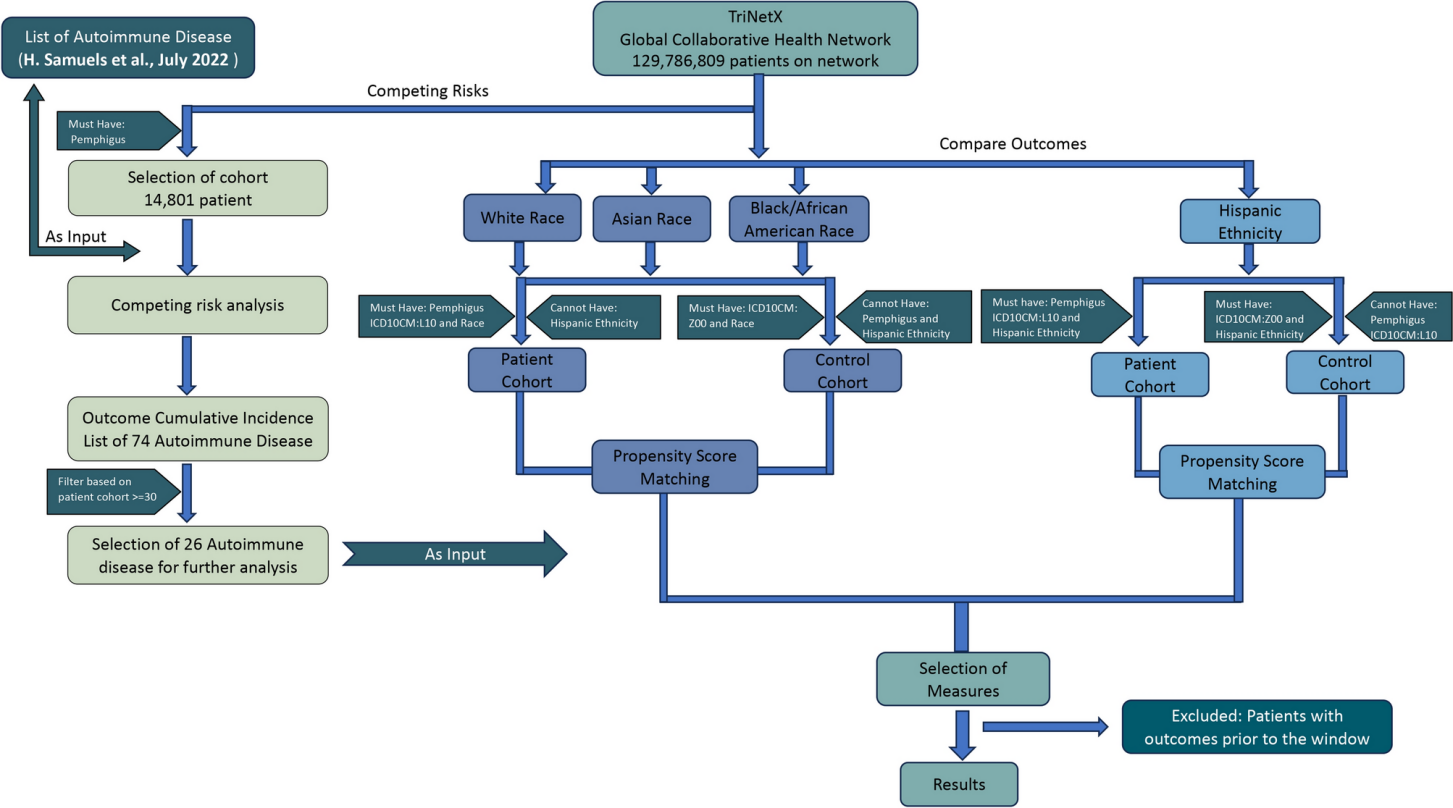
Flowchart for the study design. On the left hand the preliminary competing risks study, in which we systemically identify a comprehensive overview of autoimmune diseases with high prevalence in the studied pemphigus group. On the right hand side the compare outcomes study, in which we then compare these prevalences to a control group to measure actual comorbidity.

The study is divided into two parts to systematically explore autoimmune diseases with a high comorbidity with pemphigus (see Fig. 1). The primary analysis, termed the ’Competing Risk’ analysis, aims to identify autoimmune diseases with a high prevalence in the pemphigus group. Diseases with a patient count of at least 30 were considered for further statistical analysis in the follow-up “Compare Outcomes” study.

This secondary analysis compares pemphigus patients to a control group, with additional consideration for ethnicity-specific risk assessment. The control group, propensity-matched to the pemphigus group, was designed to control for potential confounders.

### Data set definition and propensity matching

For the initial ‘Competing Risk’ analysis, patients diagnosed with pemphigus (ICD10CM:L10) were selected from the database without consideration for sex, age, or ethnicity. Out of 108 healthcare companies worldwide, 103 provided accessible information on 129,786,809 patients, of which 17,474 were diagnosed with pemphigus. Patients with data recorded more than 20 years ago were automatically excluded by TriNetX, resulting in a final cohort of 14,801 individuals. Subsequently, this cohort underwent analysis, encompassing 92 autoimmune diseases categorized by their ICD-10-CM codes, as derived from Samuels et al.^26^. The analysis spanned from one day after the index event (diagnosis of pemphigus, ICD10CM:L10) to any time thereafter. Eighteen autoimmune diseases were excluded from the list due to various reasons: four lacked data on TriNetX, eight had uncleared ICD-10 codes, three had duplicate names, and one was restricted on TriNetX (Supplementary Table 1). After filtering, 26 diseases with at least 30 patients progressed to the follow-up study. Note that some of the diseases have slightly different names on TriNetX than in the literature, but this is noted as well on Table 1 in the supplementary.

The second “Compare Outcomes” study aimed to measure actual comorbidity by comparing pemphigus patients to a propensity-matched control group without pemphigus. Propensity matching was based on sex, age, age at index (time of pemphigus diagnosis, ICD10CM:L10), and ethnicity. In passing we note that TriNetX distinguishes between “ethnicity” and “race” technically. However, both are categorized the same and based on self-identification, so we used the terms interchangeably. The database was queried to select patients of any age and both sexes recorded on the TriNetX platform in the last 20 years, meeting the criteria for pemphigus diagnosis (ICD10CM:L10). As TriNetX limits the number of potential confounders that can be controlled simultaneously, we split the analysis into ethnicity groups, namely White, Black/African American, and Asian (Groups 1–3). Since people with a Hispanic ethnicity can belong to any or multiple of these groups, they were excluded and a Hispanic group (Group 4) was established, exclusively comprising patients of Hispanic ethnicity. Propensity score matching aligned the ethnicity-based cohorts, focusing solely on demographic criteria such as current age, age at the index (time of pemphigus diagnosis, ICD10CM:L10), and sex. Patient baseline characteristics before and after propensity score matching are shown in Table 1.

**Table 1:** Cohort characteristics before and after propensity matching.

Characteristics	Cohort	Before propensity matching	After propensity matching
White			
Current age	Patient cohort	6697	6697
	Control cohort	8,154,296	6697
Female	Patient cohort	3781	3781
	Control cohort	4,413,111	3774
Male	Patient cohort	2913	2913
	Control cohort	3,727,875	2920
Black/African American			
Current age	Patient cohort	1072	1072
	Control cohort	2,273,768	1072
Female	Patient cohort	705	705
	Control cohort	1,279,893	705
Male	Patient cohort	367	367
	Control cohort	991,386	367
Asian			
Current age	Patient cohort	1038	1038
	Control cohort	548,421	1038
Female	Patient cohort	599	599
	Control cohort	305,552	596
Male	Patient cohort	439	439
	Control cohort	242,621	442
Hispanic			
Current age	Patient cohort	1252	1252
	Control cohort	1,730,440	1252
Female	Patient cohort	677	677
	Control cohort	967,715	675
Male	Patient cohort	575	575
	Control cohort	762,099	577

Following propensity score matching, the high-prevalence diseases from the initial study were used as input. Patients experiencing the diseases before Pemphigus itself were excluded. In this analysis, we chose several measures for the comorbidity: risk ratio (RR) and odds ratio (OR) through the “Measures of Association” in TriNetX as naive direct comparisons of the prevalence in the two groups and the hazard ratio (HR) through a Kaplan-Meyer survival analysis to better take into account the risk per unit of time. Unfortunately, the OR could not be properly calculated for several subcategories due to limited data combined with the obfuscation approach used by TriNetX^27^ for patient anonymization. We base our analysis primarily on HR since this obfuscation does not apply to the hazard ratio. Furthermore, significance can only be shown up to $p<0.001$ due to platform limitations.

## Results

The demographics of the pemphigus cohort were examined to gather additional information on the minimum, maximum, and mean age, along with the standard deviation, sex, and ethnicity (see Table 2 for statistics on the tested pemphigus cohort & Fig. 3 for a visualization of the age distribution of all pemphigus patients in the TriNetX network, which is approximately comparable but not identical to our tested cohort). All age groups were included in the onset of the disease distribution, from 1 to 90 years old with an age average of 65. As expected^4^, pemphigus exhibited higher prevalence in women (56%) compared to men (43%). The majority were white (52%), while only a small portion of the cohort identified as Black/African American, Asian (8% each) or Hispanic (9%). Other ethnicities, including those categorized as just “Unknown” or “Not Hispanic or Latino”, were excluded from this study.

**Table 2:** Demographic data of the Pemphigus group.

Demographics		
Number of patients	Total patients	14,801
	Minimum age	1
	Maximum age	90
	Mean age	65
Sex	Female	56 %
	Male	43 %
	Other	1 %
Ethnicity	White	52 %
	Black/African American	8 %
	Asian	8 %
	Hispanic or Latino	9 %

Subsequently, we evaluated the risk of developing one or more of 74 distinct autoimmune diseases among pemphigus patients. The patient count, percentage of each cohort, and cumulative incidence after a specified time window (from one day after the diagnosis of pemphigus, ICD10CM:L10, to any subsequent time) are outlined in Supplementary Table 2. Bullous pemphigoid had the highest patient count (1535), followed by cicatricial pemphigoid (438), psoriasis (424), and (other) rheumatoid arthritis (356). Conversely, diseases with the smallest patient counts included eosinophilic esophagitis (31), sarcoidosis (36), alopecia areata (39), and immune thrombocytopenic purpura (41), offering insight into the distribution of cases within the examined cohorts. We defined comorbidity through the variable “Cumulative incidence at the end of the time window’. It refers to the number of individuals experiencing a specific event by the conclusion of a defined time frame. In our study, this window extends from the day following the index event to any subsequent time thereafter. The findings indicate that bullous pemphigoid (13.57%), (other) rheumatoid arthritis (6.77%), psoriasis (6.44%), and cicatricial pemphigoid (5.59%) exhibit the highest occurrence rates following pemphigus.

To minimize bias stemming from confounding factors, we conducted propensity matching as outlined in the “Methods” section. Supplementary Table 3 presents the baseline characteristics of patients before and after propensity score matching. Overall, there is considerable variation in ethnicity frequencies across most categories, highlighting the necessity of separately considering these groups. Particularly noteworthy is the observation that Hispanic and white ethnicities represent the youngest and oldest groups, respectively, with the earliest and latest onset of disease ($46.9\pm 19.3$; $P\le 0.001$ vs. $62.1\pm 18.3$; $P\le 0.001$), which might stem from the large change in the Hispanics demographics within the United States of America during the last 20 years. Furthermore, although all groups exhibit a sex bias favoring women, the Black group demonstrates the highest female predominance, with 705 patients (65.8%) compared to 367 male patients (34.2%).

The analysis comparing outcomes reveals the risk of developing comorbid autoimmune diseases among patients diagnosed with pemphigus, delineated by ethnicity, as presented in Supplementary Table 4–7. Pemphigus patients of white ethnicity exhibited a significantly elevated risk of developing bullous pemphigoid [HR 145.343; OR 57.347; $P\le 0.001$]. Moreover for this group, a significantly increased risk was noted for lichen planus [HR 6.477; OR 5.993; $P\le 0.001$], systemic involvement of connective tissue [HR 4.614; OR 4.250; $P\le 0.001$], discoid lupus erythematosus [HR 3.912; OR 3.632; $P\le 0.001$], antiphospholipid syndrome [HR 3.287; OR 1.504; $P=0.015$], and myasthenia gravis [HR 2.893; OR 1.603; $P=0.02$]. Notably, cicatricial pemphigoid exhibited the most pronounced and significant risk [HR 222.167; OR 20.728; $P\le 0.001$] for the white group. Additionally, a significant risk of other autoimmune diseases, including systemic lupus erythematosus, neutropenia, Sjögren’s syndrome, other rheumatoid arthritis, and psoriasis, was identified.

On the other hand, black individuals diagnosed with pemphigus exhibited a significantly higher risk of developing lichen planus [HR 12.723; OR 1.217; $P=0.002$] and discoid lupus erythematosus [HR 10.176; OR 1.012; $P=0.006$] compared to their counterparts in the white group. Notably, among black pemphigus patients, the highest risk was observed for thyrotoxicosis with diffuse goiter [HR 9.740; OR 1.005; $P=0.008$] compared to other ethnic groups. Similarly, elevated risks were noted for psoriasis [HR 3.864; OR 1.760; P = 0.004] and rheumatoid arthritis [HR 2.595; OR 2.076; $P=0.014$].

Similarly, Hispanic patients diagnosed with pemphigus showed an increased risk of developing lichen planus [HR 16.083; OR 1.415; $P\le 0.001$] and discoid lupus erythematosus [HR 6.418; OR 1.208; $P=0.005$]. Elevated risks were also observed for other rheumatoid arthritis [HR 7.065; OR 3.101; $P\le 0.001$] and Sjögren’s syndrome [HR 4.838; OR 1.313; $P=0.007$]. Interestingly, among Asian pemphigus patients, the highest risks were identified for systemic lupus erythematosus [HR 20.460; OR 3.977; $P\le 0.001$] and antiphospholipid syndrome [HR 16.869; OR 1.513; $P\le 0.001$]. In contrast, a comparatively lower risk was observed for lichen planus [HR 8.466; OR 1.655; $P=0.001$], psoriasis [HR 6.596; OR 4.527; $P\le 0.001$], and Sjögren’s syndrome [HR 6.187; OR 5.690; $P\le 0.001$] among Asian pemphigus patients.

**Figure 2:**
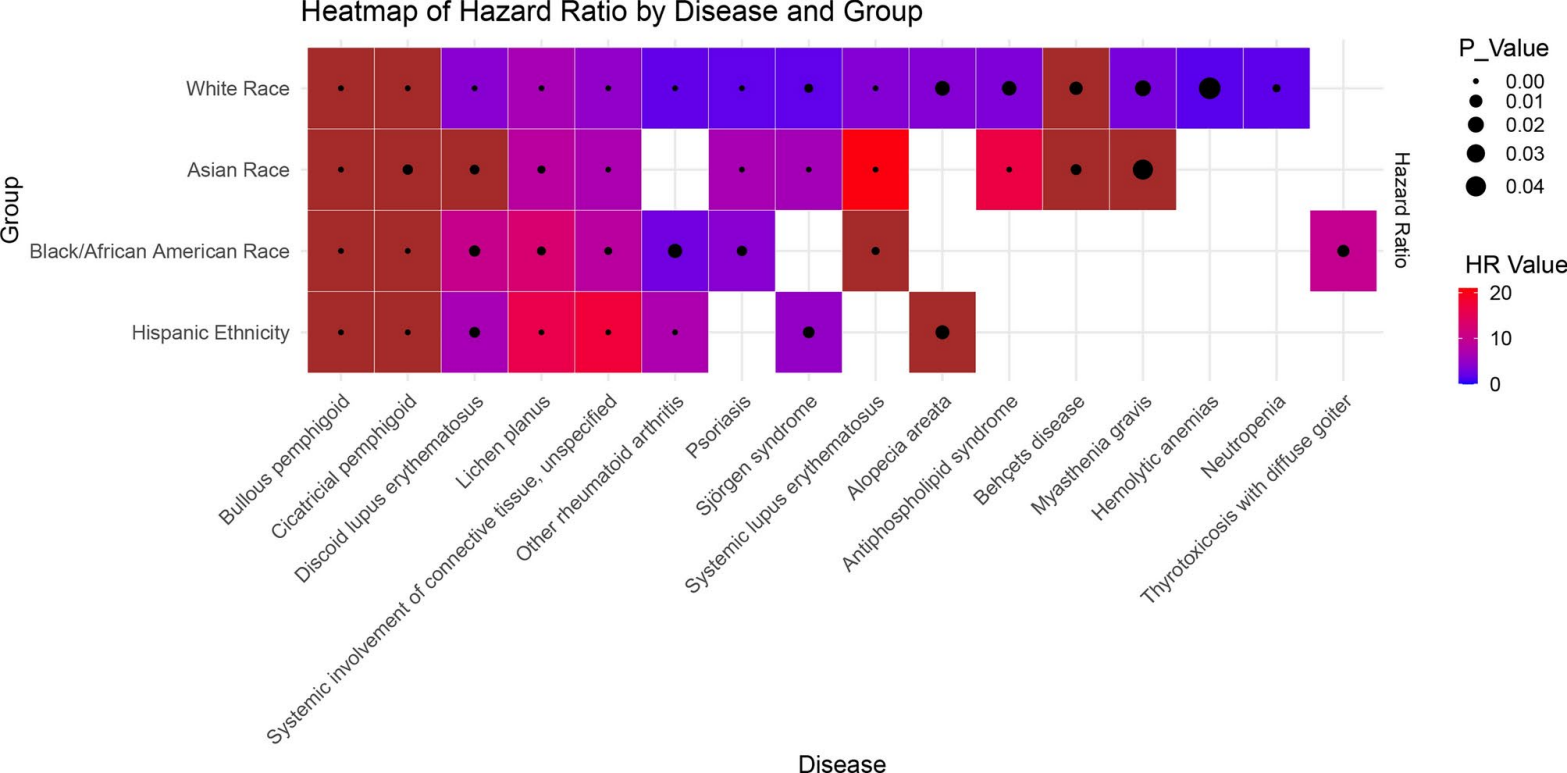
Comorbid autoimmune diseases with a significantly elevated HR in at least one group. The color indicates the strength of the increase, the size of the black dot indicates the degree of significance. Only significant relationships are shown.

As depicted in Fig. 2, individuals diagnosed with pemphigus manifested a significantly heightened susceptibility to developing bullous pemphigoid and cicatricial pemphigoid across all ethnic groups. A similar trend was observed for other diseases, with elevated prevalence among pemphigus patients in most subgroups, although statistical significance may not be reached due to limited data. In the White group, only two diseases had fewer than 10 patients available, while the highest number of diseases was observed among Asian patients (15 diseases), with both the Black and Hispanic groups having 12 diseases each. Notably, individuals across all ethnic backgrounds demonstrated a significantly increased likelihood of developing discoid lupus erythematosus, lichen planus, and systemic involvement of connective tissue. Antiphospholipid syndrome exhibited significance among White and Asian populations, whereas psoriasis showed increased prevalence in all groups, although statistical significance was not reached in Hispanics. Myasthenia gravis was found to be significant in White and Asian populations but not in the other two groups.

**Figure 3:**
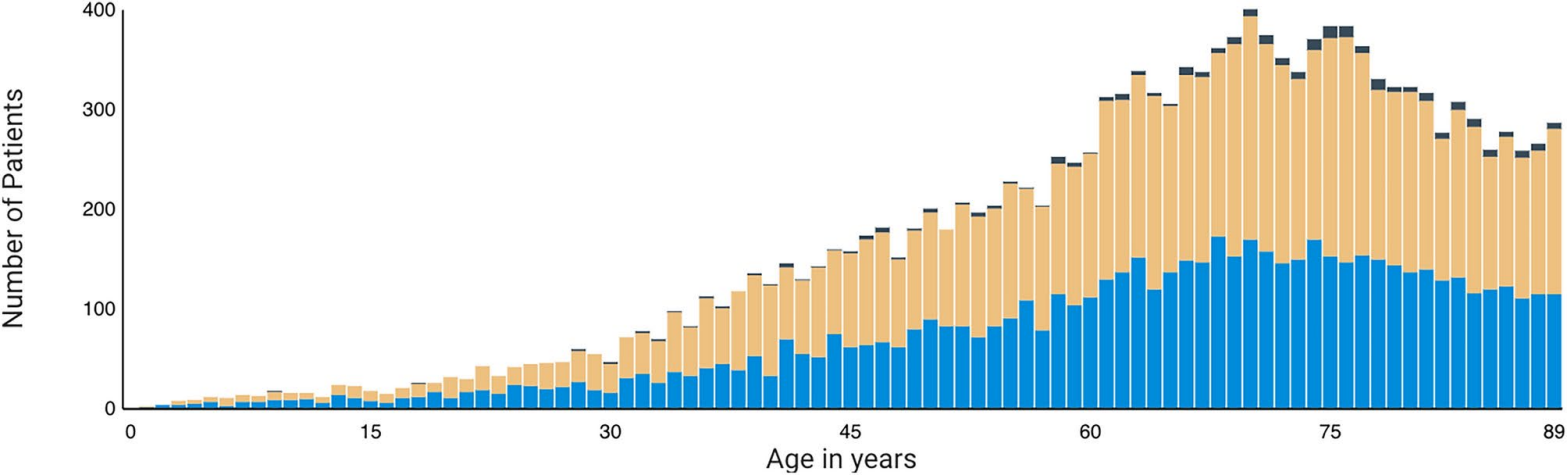
Age distribution of all patients with pemphigus on TriNetX as of 9.10.2024. Note that the hard cutoff at 90 is due to TriNetX’ data obfuscation approach.

## Discussion

Several investigations have suggested that individuals diagnosed with pemphigus are predisposed to developing concurrent autoimmune disorders, including psoriasis, various forms of rheumatoid arthritis, lichen planus, Sjörgen syndrome, systemic lupus erythematosus, and alopecia areata^21,28^. Comparable patterns were observed within the White group; however, statistical significance was not reached for Sjörgen syndrome and alopecia areata in the Black group, and for other forms of rheumatoid arthritis and alopecia areata in the Asian group. Similarly, in the Hispanic group, psoriasis and systemic lupus erythematosus did not demonstrate significance. Since almost all of these relationships show a consistent trend across all categories, merely not reaching significance in some subgroups, they are plausibly an issue of limited data. While Kridin et al., reported an association between ulcerative colitis and pemphigus, but not Crohn’s disease, our study did not find any correlation between pemphigus and either ulcerative colitis or Crohn’s disease in any ethnic group. Both of these diseases are variants of inflammatory bowel diseases and may not even be autoimmune, mereley immune system related^29^, and as a result a lack of relationship with pemphigus is quite plausible. Additionally, no associations were found with autoimmune thyroiditis, celiac disease, eosinophilic esophagitis, immune thrombocytopenic purpura, or sarcoidosis. Multiple sclerosis was not linked with pemphigus in previous studies, and our analysis revealed the absence of association across all ethnic groups^30^. Particularly, no evidence of anti-correlations or protective effects between the investigated autoimmune diseases was found.

Our findings hold significant relevance for clinical practice, underscoring the necessity of screening for comorbidities to ensure comprehensive care for pemphigus patients. Many of the most prominent comorbid autoimmune diseases affect the skin, which could be overlooked by physicians when a patient already presents with pemphigus. Among the strongest non-pemphigoid diseases are discoid and systemic lupus erythematosus. Both of these can add further complications that need to be considered, such as photosensitivity, renal or blood disorders and which might require an increase in immunosuppressive drug doses such as prednisonone^31^. These two further belong to a large cluster of autoimmune connective tissue diseases associated with pemphigus also including rheumatoid arthritis, Sjörgen and undifferentiated connective tissue disease, which is named as “Systemic involvement of connective tissue, unspecified” in TriNetX. An exhaustive list of possible complications and further required treatment is not feasible here, but it includes among others joint problems requiring physiotherapy, dryness of soft tissues which preclude certain medications, stress triggers requiring psychotherapy or complications between the osteoporosis caused by autoimmune symptoms and glucosteroids.

Conversely, certain associations, such as those with antiphospholipid syndrome, neutropenia, and thyroid toxicosis, may come as unexpected. To date, evidence of elevated levels of antiphospholipid antibodies in pemphigus patients has been limited to a small study^32^. While prior theories have suggested a potential link between neutropenia and pemphigus treatment with immunosuppressives, investigations have shown a low prevalence^33^.

Much of the existing research has primarily focused on single-institutional studies, leaving a gap for a comprehensive, multi-institutional investigation on a global scale. Our study aimed to fill this gap by conducting a detailed quantitative analysis, with a particular emphasis on the influence of race and ethnicity on the susceptibility of pemphigus patients to secondary autoimmune diseases, which may manifest as comorbidities. The investigation thoroughly examined the risk of secondary autoimmune diseases among pemphigus patients across diverse racial and ethnic groups. The results consistently demonstrated robust associations between pemphigus and other autoimmune conditions, notably pemphigoid.

Our discoveries are broadly consistent with previous research indicating a correlation between various autoimmune disorders^8,10^. In comparison to another recent review utilizing TriNetX data^34^, our findings reveal similar associations among autoimmune diseases, despite some notable methodological distinctions. While Kasperskiewicz et al., additionally adjusted for obesity and nicotine dependence and separately examined associations with Bullous Pemphigoid, they analyzed a smaller cohort of Pemphigus patients ($n \sim 8600$), a reduced subset of 25 autoimmune disorders, and only differentiated between White and Black/African American racial categories, excluding Asians and Hispanics. Additionally, a minor issue observed in their supplementary materials is the inadequate exclusion of group sizes with $n\le 10$, which could lead to both false positives and false negatives. For instance, in supplementary table 9 of Kasperskiewicz et al.^34^, the risk difference (0.272, line 2) may be inflated due to rounding up in the case group, while the risk difference (0.055, line 3) may be deflated due to rounding up in the control group. On line 4, the direction of bias is indeterminable since both sides are rounded. The last case is the most common on TriNetX, and as a result there is a notable bias towards insignificance for rarer states on the platform if not controlled for. In contrast, our study excludes odds ratios (OR) and related measures such as risk difference if any value is 10 or less to mitigate this potential error.

This study possesses several limitations that warrant consideration. Firstly, reliance on electronic health record data introduces potential biases stemming from misdiagnosis and inaccurate coding, which can compromise data precision. This applies in particular to diseases with a similar presentation, such as pemphigus and the pemphigoid diseases. Secondly, our analysis is confined to patients with access to healthcare services, thereby excluding those lacking such access, which may constrain the generalizability of our findings. Thirdly, our results are contingent upon the data available within TriNetX at the time of analysis, precluding the possibility of rerunning the analysis with identical patient numbers at a later time. Moreover, the number of covariates available for propensity matching is limited. In our study, the large pemphigus cohort necessitated segmentation into racial and ethnic groups to facilitate accurate matching. While we excluded data entries labeled as “unknown” for race, ethnicity, and sex, this does not entirely mitigate biases. Additionally, constraints such as raw data unavailability on TriNetX and limitations in statistical methods impeded more sophisticated analyses. Given the rarity of the disease and limited patient counts, particularly among non-White ethnicities, statistics derived from such small samples may be unreliable or unattainable. Furthermore, TriNetX employs a practice of rounding small patient numbers to 10 to protect patient anonymity, exacerbating the challenge of rarity and resulting in control subgroups with zero disease cases, rendering the calculation of hazard ratios (HR) and odds ratios (OR) infeasible despite statistical significance^27^. Lastly, establishing a causal relationship is challenging in epidemiological data, particularly regarding the direction of causality. While we excluded patients who developed one of the tested diseases before being diagnosed with pemphigus, this precaution alone may not be sufficient to confidently establish a causal link.

Despite these limitations, our study possesses significant strengths, particularly its expansive sample size and diverse patient demographics. As illustrated in Table 1, the large dataset facilitates robust propensity matching between pemphigus and non-pemphigus groups, thereby minimizing errors arising from disparate characteristics such as age. By harnessing data from a comprehensive database, many of our findings are representative and applicable across diverse populations.

Notwithstanding the strides in global collaboration enabling the aggregation of much larger datasets, the available data remains inadequate, underscoring the need for further comprehensive research to glean essential insights. Additional experimental investigations are imperative to elucidate the underlying pathomechanisms of these comorbidities in pemphigus patients.

## Electronic supplementary material

Below is the link to the electronic supplementary material.

Supplementary Material 1

## Data Availability

Access to the TriNetX research network is granted through a request to TriNetX (https://live.trinetx.com). Only anonymized patient data is available. This requires a data sharing agreement and may incur costs. Full analysis data available on reasonable request from the corresponding author.
