# Supplementary material for: Large-scale global retrospective study on the interaction between ancestry and risk of comorbid autoimmune diseases in patients with pemphigus
Source: Sci Rep. 2024 Dec 3;14:30151. doi: 10.1038/s41598-024-78031-z (PMC11614865; doi:10.1038/s41598-024-78031-z)
Supplement: Supplementary file 1 — Supplementary Material 1 [file 41598_2024_78031_MOESM1_ESM.pdf]

Supplementary Table 1 : An inventory of 93 autoimmune diseases organized by their corresponding ICD-10-CM codes. The first column enumerates the diseases identified from literature, the second column specifies the affected system, the third column contains the ICD-10 codes, and the fourth column provides the corresponding terms found on TriNetX using the respective ICD codes.			
**Autoimmune Disease In Litrature (Samuels et. al.)**	**Affected System**	**ICD-10 Code**	**Autoimmune Disease Name On TriNetX**
Addison’s disease	Endrocrine	G73.7	Myopathy in diseases classified elsewhere
Alopecia areata	Cutaneous	L63	Alopecia areata
Amyloidosis	Multiple	E85	Amyloidosis
Antiphospholipid syndrome	Hematopoietic	D68.61	Antiphospholipid syndrome
Autoimmune hepatitis type 1	Gastrointestinal	K75.4	Autoimmune hepatitis
Bullous pemphigoid	Cutaneous	L12.0	Bullous pemphigoid
Castleman disease	Multiple	D47.Z2	Castleman disease
Celiac disease	Gastrointestinal	K90.0	Celiac disease
Chronic inflammatory demyelinating polyneuropathy	Neuronal	G61.81	Chronic inflammatory demyelinating polyneuritis
Cicatricial pemphigoid	Cutaneous	L12.1	Cicatricial pemphigoid
Cogan’s syndrome	Multiple	H16.32	Diffuse interstitial keratitis
Cold agglutinin disease	Hematopoietic	D59.12	Cold autoimmune hemolytic anemia
Congenital heart block	Cardiovascular	Q24.6	Congenital heart block
CREST syndrome	Multiple	M34.1	Cr(e)st syndrome
Crohn’s disease	Gastrointestinal	K50	Crohn's disease [regional enteritis]
Dermatitis herpetiformis	Cutaneous	L13.0	Dermatitis herpetiformis
Dermatomyositis	Musculoskeletal	M33.1	Other dermatomyositis
Discoid lupus erythematosus	Cutaneous	L93.0	Discoid lupus erythematosus
Eosinophilic esophagitis	Gastrointestinal	K20.0	Eosinophilic esophagitis
Eosinophilic fasciitis	Cutaneous	M35.4	Diffuse (eosinophilic) fasciitis
Evans syndrome	Hematopoietic	D69.41	Evans syndrome
Felty’s syndrome	Hematopoietic	M05.0	Felty's syndrome
Fibrosing alveolitis	Kidneys and Lungs	J84.112	Idiopathic pulmonary fibrosis
Goodpasture’s disease	Kidneys and Lungs	M31.0	Hypersensitivity angiitis
Granulomatosis with polyangiitis	Cardiovascular	M31.3	Wegener's granulomatosis
Graves’ disease	Endrocrine	E05.0	Thyrotoxicosis with diffuse goiter
Guillain–Barré Syndrome	Neuronal	G61.0	Guillain-barre syndrome
Hashimoto’s thyroiditis	Endrocrine	E06.3	Autoimmune thyroiditis
Hemolytic anemia	Hematopoietic	D55-D59	Hemolytic anemias
Inclusion body myositis	Musculoskeletal	G72.41	Inclusion body myositis [ibm]
Kawasaki disease	Cardiovascular	M30.3	Mucocutaneous lymph node syndrome [kawasaki]
Lichen planus	Cutaneous	L43	Lichen planus
Microscopic polyangiitis	Cardiovascular	M31.7	Microscopic polyangiitis
Mucha–Habermann disease	Cutaneous	L41.0	Pityriasis lichenoides et varioliformis acuta
Multifocal Motor Neuropathy	Neuronal	G61.82	Multifocal motor neuropathy
Multiple sclerosis	Neuronal	G35	Multiple sclerosis

Myasthenia gravis	Neuronal	G70.0	Myasthenia gravis
Narcolepsy	Neuronal	G47.41	Narcolepsy
Neuromyelitis optica	Neuronal	G36.0	Neuromyelitis optica [devic]
Neutropenia	Hematopoietic	D70	Neutropenia
Optic neuritis	Neuronal	H46	Optic neuritis
Paroxysmal nocturnal hemoglobinuria	Hematopoietic	D59.5	Paroxysmal nocturnal hemoglobinuria [marchiafava-micheli]
Parry Romberg syndrome	Cutaneous	G51.8	Other disorders of facial nerve
Pernicious anemia		D51.0	Vitamin b12 deficiency anemia due to intrinsic factor deficiency
Polyarteritis nodosa	Cardiovascular	M30.0	Polyarteritis nodosa
Polyglandular syndrome 2	Endrocrine	E31.0	Autoimmune polyglandular failure
Polymyositis	Musculoskeletal	M33.2	Polymyositis
Primary biliary cholangitis	Gastrointestinal	K74.3	Primary biliary cirrhosis
Primary sclerosing cholangitis	Gastrointestinal	K83.01	Primary sclerosing cholangitis
Psoriasis	Cutaneous	L40	Psoriasis
Relapsing polychondritis	Musculoskeletal	M94.1	Relapsing polychondritis
Rheumatoid arthritis	Multiple	M06	Other rheumatoid arthritis
Sarcoidosis	Multiple	D86	Sarcoidosis
Scleritis	Eye	H15.0	Scleritis
Scleroderma	Multiple	M34	Systemic sclerosis [scleroderma]
Sjögren’s syndrome	Multiple	M35.0	Sjögren syndrome
Stiff person syndrome	Neuronal	G25.82	Stiff-man syndrome
Still’s disease	Multiple	M06.1	Adult-onset still's disease
Susac syndrome	Cardiovascular	I67.7	Cerebral arteritis, not elsewhere classified
Sympathetic ophthalmia	Eye	H44.13	Sympathetic uveitis
Systemic lupus erythematosus	Multiple	M32	Systemic lupus erythematosus (sle)
Takayasu arteritis	Cardiovascular	M31.4	Aortic arch syndrome [takayasu]
Thrombocytopenic purpura	Hematopoietic	D69.3	Immune thrombocytopenic purpura
Type 1 diabetes mellitus	Endrocrine	E10	Type 1 diabetes mellitus
Ulcerative colitis	Gastrointestinal	K51	Ulcerative colitis
Uveitis (Behçet’s disease)	Eye	M35.2	Behçet's disease
Vitiligo	Cutaneous	L80	Vitiligo
Mixed connective tissue disease	Multiple	M35.9	Systemic involvement of connective tissue, unspecified
Lambert–Eaton syndrome	Neuronal	G70.80	Lambert-eaton syndrome, unspecified
Neonatal Lupus	Cardiovascular	P37.5	TriNetX showed term “restricted”
Temporal arteritis	Cardiovascular	?	The ICD-10 code was not detected.
Alzheimer’s disease (autoimmune caused)	Neuronal	?	The ICD-10 code was not detected.
Autoimmune inner ear disease	Neuronal	H83.8X9	Termed as Other specified diseases of inner ear, unspecified ear
Autoimmune oophoritis	Endrocrine	N70.92	Oophoritis unspecified
Autoimmune orchitis	Endrocrine	N45.9	not available on TriNetX
Autoimmune pancreatitis	Multiple	?	The ICD-10 code was not detected.
Glomerulonephritis	Kidneys	?	The ICD-10 code was not detected.
Hashimoto’s encephalopathy	Neuronal	G04.81	Termed as Other encephalitis and encephalomyelitis
POEMS syndrome	Multiple	D47.7	Not available on TriNetX
Polyglandular syndrome 1	Endrocrine	O35.14X1	Termed as Maternal care for (suspected) chromosomal abnormality in fetus, Turner Syndrome, fetus 1

Polyglandular syndrome 3	Endrocrine	O35.14X3	not available on TriNetX
Progesterone dermatitis	Cutaneous	?	The ICD-10 code was not detected.
Retroperitoneal fibrosis	Multiple	K68.2	Not available on TriNetX
Thyroid eye disease	Eye	?	The ICD-10 code was not detected.
Transverse myelitis	Neuronal	G37.3	Termed as Acute transverse myelitis in demyelinating disease of central nervous system
Vasculitis	Cardiovascular	?	The ICD-10 code was not detected.
Vogt–Koyanagi–Harada Disease	Eye, Neuronal	?	The ICD-10 code was not detected.
Pemphigus	Cutaneous	L10	Excluded
Pemphigus foliaceus	Cutaneous	L10.2	Excluded
Undifferentiated connective tissue disease	Cutaneous	M35.9	Termed as Systemic involvement of connective tissue, unspecified
Miller Fisher syndrome	Neuronal	G61.0	Termed as Guillain-Barre syndrome
Autoimmune hepatitis type 2	Gastrointestinal	K75.4	Termed as Autoimmune hepatitis

Supplementary Table 2 : The table presents data regarding the patient count, the percentage of each cohort, and the cumulative incidence at the end of the specified time window. The gray highlighted section indicates diseases with patient counts exceeding 30.

Outcome Name	Patient Count	Percentage of Cohort	Cumulative Incidence at End of Time Window
Bullous pemphigoid	1535	10,97 %	13,57 %
Cicatricial pemphigoid	438	3,13 %	5,59 %
Psoriasis	424	3,03 %	6,44 %
Other rheumatoid arthritis	356	2,54 %	6,77 %
Sjögren syndrome	343	2,45 %	4,95 %
Lichen planus	231	1,65 %	3,35 %
Systemic lupus erythematosus (sle)	229	1,64 %	3,41 %
Neutropenia	205	1,46 %	4,04 %
Systemic involvement of connective tissue, unspecified	188	1,34 %	3,60 %
Type 1 diabetes mellitus	133	0,95 %	3,80 %
Discoid lupus erythematosus	115	0,82 %	1,79 %
Hemolytic anemias	111	0,79 %	4,00 %
Ulcerative colitis	86	0,61 %	2,14 %
Behçet's disease	76	0,54 %	0,72 %
Crohn's disease [regional enteritis]	75	0,54 %	1,09 %
Autoimmune thyroiditis	70	0,50 %	0,96 %
Thyrotoxicosis with diffuse goiter	65	0,46 %	1,10 %
Multiple sclerosis	65	0,46 %	0,72 %
Antiphospholipid syndrome	55	0,39 %	1,37 %
Myasthenia gravis	50	0,36 %	0,64 %
Celiac disease	48	0,34 %	0,57 %
Vitiligo	45	0,32 %	0,71 %
Immune thrombocytopenic purpura	41	0,29 %	0,86 %
Alopecia areata	39	0,28 %	0,66 %
Sarcoidosis	36	0,26 %	0,47 %
Eosinophilic esophagitis	31	0,22 %	0,60 %
Scleritis	26	0,19 %	0,33 %
Amyloidosis	25	0,18 %	0,80 %
Dermatitis herpetiformis	25	0,18 %	0,34 %
Vitamin b12 deficiency anemia due to intrinsic factor deficiency	24	0,17 %	0,36 %
Idiopathic pulmonary fibrosis	23	0,16 %	0,43 %
Hypersensitivity angiitis	20	0,14 %	0,45 %
Autoimmune hepatitis	20	0,14 %	0,42 %
Optic neuritis	18	0,13 %	0,42 %
Castleman disease	17	0,12 %	0,16 %
Chronic inflammatory demyelinating polyneuritis	16	0,11 %	0,20 %
Wegener's granulomatosis	14	0,10 %	0,45 %
Cr(e)st syndrome	13	0,09 %	0,13 %
Systemic sclerosis [scleroderma]	11	0,08 %	0,33 %
Other disorders of facial nerve	10	0,07 %	0,28 %

**Other dermatomyositis**	10	0,07 %	0,26 %
**Primary biliary cirrhosis**	10	0,07 %	0,23 %
**Neuromyelitis optica [devic]**	10	0,07 %	0,19 %
**Primary sclerosing cholangitis**	10	0,07 %	0,14 %
**Pityriasis lichenoides et varioliformis acuta**	10	0,07 %	0,14 %
**Narcolepsy**	10	0,07 %	0,12 %
**Other encephalitis and encephalomyelitis**	10	0,07 %	0,09 %
**Polymyositis**	10	0,07 %	0,09 %
**Guillain-barre syndrome**	10	0,07 %	0,08 %
**Cerebral arteritis, not elsewhere classified**	10	0,07 %	0,07 %
**Cold autoimmune hemolytic anemia**	10	0,07 %	0,06 %
**Relapsing polychondritis**	10	0,07 %	0,06 %
**Autoimmune polyglandular failure**	10	0,07 %	0,06 %
**Lambert-eaton syndrome, unspecified**	10	0,07 %	0,06 %
**Diffuse (eosinophilic) fasciitis**	10	0,07 %	0,05 %
**Polyarteritis nodosa**	10	0,07 %	0,03 %
**Oophoritis, unspecified**	10	0,07 %	0,03 %
**Adult-onset still's disease**	10	0,07 %	0,03 %
**Inclusion body myositis [ibm]**	10	0,07 %	0,03 %
**Sympathetic uveitis**	10	0,07 %	0,03 %
**Acute transverse myelitis in demyelinating disease of central nervous system**	10	0,07 %	0,02 %
**Felty's syndrome**	10	0,07 %	0,02 %
**Aortic arch syndrome [takayasu]**	10	0,07 %	0,02 %
**Diffuse interstitial keratitis**	10	0,07 %	0,02 %
**Microscopic polyangiitis**	10	0,07 %	0,01 %
**Stiff-man syndrome**	10	0,07 %	0,01 %
**Mucocutaneous lymph node syndrome [kawasaki]**	10	0,07 %	0,01 %
**Multifocal motor neuropathy**	10	0,07 %	0,01 %
**Myopathy in diseases classified elsewhere**	10	0,07 %	0,01 %
**Other specified diseases of inner ear, unspecified ear**	10	0,07 %	0,01 %
**Evans syndrome**	10	0,07 %	0,01 %
**Congenital heart block**	0	0,00 %	0,00 %
**Paroxysmal nocturnal hemoglobinuria [marchiafava-micheli]**	0	0,00 %	0,00 %
**Maternal care for (suspected) chromosomal abnormality in fetus, turner syndrome, fetus 1**	0	0,00 %	0,00 %

Supplementary Table 3 : Cohort Characteristics before and after propensity matching. This table comprises characteristics such as mean and standard deviation of current age and age at index, count of patient by gender, data regarding the percentage of the cohort, P-values, and standardized mean differences.

			Before Matching					After Matching				
**Cohort**		**Characteristics**	**Mean ± SD**	**Patients**	**% of Cohort**	**P-Value**	**Std diff.**	**Mean ± SD**	**Patients**	**% of Cohort**	**P-Value**	**Std diff.**
		**White Race**										
Patient Cohort	Age	Current Age	68.4 +/- 17.8	6697	100 %	<0,001	1,024	68.4 +/- 17.8	6697	100 %	0,997	<0,001
Control Cohort			46.4 +/- 24.7	8154296	100 %			68.4 +/- 17.8	6697	100 %		
Patient Cohort	AI	Age at Index	62.1 +/- 18.3	6697	100 %	<0,001	1,01	62.1 +/- 18.3	6697	100 %	0,972	0,001
Control Cohort			40.1 +/- 24.8	8154296	100 %			62.1 +/- 18.4	6697	100 %		
Patient Cohort	F	Female		3781	56,50 %	<0,001	0,047		3781	56,50 %	0,903	0,002
Control Cohort				4413111	54,10 %				3774	56,40 %		
Patient Cohort	M	Male		2913	43,50 %	<0,001	0,045		2913	43,50 %	0,903	0,002
Control Cohort				3727875	45,70 %				2920	43,60 %		
Patient Cohort	2106-3	White		6697	100 %	--	--		6697	100 %	--	--
Control Cohort				8154296	100 %				6697	100 %		
		**Black/African American Race**										
Patient Cohort	Age	Current Age	62.1 +/- 18.3	1072	100 %	<0,001	1,193	62.1 +/- 18.3	1072	100 %	0,995	<0,001
Control Cohort			36.5 +/- 24.2	2273768	100 %			62.1 +/- 18.3	1072	100 %		
Patient Cohort	AI	Age at Index	55.9 +/- 18.8	1072	100 %	<0,001	1,188	55.9 +/- 18.8	1072	100 %	0,994	<0,001
Control Cohort			30.0 +/- 24.4	2273768	100 %			55.9 +/- 18.8	1072	100 %		
Patient Cohort	F	Female		705	65,80 %	<0,001	0,195		705	65,80 %	1	<0,001
Control Cohort				1279893	56,30 %				705	65,80 %		
Patient Cohort	M	Male		367	34,20 %	<0,001	0,193		367	34,20 %	1	<0,001
Control Cohort				991386	43,60 %				367	34,20 %		
Patient Cohort	2054-5	Black or African American		1072	100 %	--	--		1072	100 %	--	--
Control Cohort				2273768	100 %				1072	100 %		
		**Asian Race**										
Patient Cohort	Age	Current Age	59.6 +/- 17.7	1038	100 %	<0,001	1,104	59.6 +/- 17.7	1038	100 %	0,953	0,003
Control Cohort			37.0 +/- 22.9	548421	100 %			59.7 +/- 17.8	1038	100 %		
Patient Cohort	AI	Age at Index	52.3 +/- 18.2	1038	100 %	<0,001	1,029	52.3 +/- 18.2	1038	100 %	0,933	0,004
Control Cohort			31.0 +/- 22.9	548421	100 %			52.3 +/- 18.2	1038	100 %		
Patient Cohort	F	Female		599	57,70 %	0,197	0,04		599	57,70 %	0,894	0,006
Control Cohort				305552	55,70 %				596	57,40 %		
Patient Cohort	M	Male		439	42,30 %	0,207	0,039		439	42,30 %	0,894	0,006
Control Cohort				242621	44,20 %				442	42,60 %		
Patient Cohort	2028-9	Asian		1038	100 %	--	--		1038	100 %	--	--
Control Cohort				548421	100 %				1038	100 %		
		**Hispanic Ethnicity**										
Patient Cohort	Age	Current Age	53.5 +/- 19.4	1252	100 %	<0,001	1,203	53.5 +/- 19.4	1252	100 %	0,949	0,003
Control Cohort			28.9 +/- 21.4	1730440	100 %			53.4 +/- 19.5	1252	100 %		
Patient Cohort	AI	Age at Index	46.9 +/- 19.3	1252	100 %	<0,001	1,23	46.9 +/- 19.3	1252	100 %	0,958	0,002
Control Cohort			21.9 +/- 21.4	1730440	100 %			46.9 +/- 19.4	1252	100 %		

Patient Cohort	F	Female		677	54,10 %	0,188	0,037		677	54,10 %	0,936	0,003
Control Cohort				967715	55,90 %				675	53,90 %		
Patient Cohort	M	Male		575	45,90 %	0,179	0,038		575	45,90 %	0,936	0,003
Control Cohort				762099	44,00 %				577	46,10 %		
Patient Cohort	2106-3	White		723	57,70 %	0,06	0,053		723	57,70 %	0,872	0,006
Control Cohort				1044230	60,30 %				719	57,40 %		
Patient Cohort	2054-5	Black or African American		13	1,00 %	0,002	0,102		13	1,00 %	0,847	0,008
Control Cohort				40852	2,40 %				14	1,10 %		
Patient Cohort	1002-5	American Indian or Alaska Native		10	0,80 %	0,199	0,033		10	0,80 %	1	<0,001
Control Cohort				9245	0,50 %				10	0,80 %		
Patient Cohort	2028-9	Asian		10	0,80 %	0,056	0,046		10	0,80 %	1	<0,001
Control Cohort				7633	0,40 %				10	0,80 %		

Supplementary Table 4 : White Group Analysis						
**Outcome Name**	**Patients in cohort**	**Patients with outcome**	**OR (95% CI)**	**P-value**	**HR (95% CI)**	**Log- Rank P-value**
Alopecia areata	6687	13	1.302 (0.570, 2.971)	0,530	3.631 (1.184, 11.138)	0,016
Antiphospholipid syndrome	6666	15	1.504 (0.675, 3.351)	0,314	3.287 (1.195, 9.045)	0,015
Autoimmune thyroiditis	6647	38	0.791 (0.516, 1.213)	0,281	0.867 (0.567, 1.327)	0,511
Behçet’s disease	6681	≤ 10 *	--	--	--	0,011
Bullous pemphigoid	5721	452	57.347 (30.614, 107.424)	<0,001	145.343 (54.314, 388.940)	<0,001
Celiac disease	6659	17	1.705 (0.780, 3.737)	0,176	2.062 (0.919, 4.626)	0,073
Cicatricial pemphigoid	6386	192	20.728 (10.966, 39.180)	<0,001	222.167 (31.135, 1585.276)	<0,001
Crohn’s disease [regional enteritis]	6646	21	1.171 (0.623, 2.200)	0,623	1.273 (0.678, 2.389)	0,452
Discoid lupus erythematosus	6661	36	3.632 (1.801, 7.324)	<0,001	3.912 (1.942, 7.884)	<0,001
Eosinophilic esophagitis	6681	15	1.504 (0.675, 3.352)	0,314	1.808 (0.791, 4.132)	0,154
Hemolytic anemias	6651	45	1.460 (0.923, 2.309)	0,104	1.579 (0.999, 2.495)	0,049
Immune thrombocytopenic purpura	6671	15	1.255 (0.587, 2.683)	0,558	1.358 (0.635, 2.901)	0,428
Lichen planus	6567	70	5.993 (3.246, 11.067)	0,000	6.477 (3.510, 11.949)	<0,001
Multiple sclerosis	6651	16	1.337 (0.632, 2.829)	0,446	1.462 (0.692, 3.092)	0,317
Myasthenia gravis	6678	16	1.603 (0.727, 3.536)	0,238	2.893 (1.132, 7.393)	0,020
Neutropenia	6600	97	1.589 (1.153, 2.189)	0,004	1.718 (1.249, 2.363)	0,001
Other rheumatoid arthritis	6498	105	1.760 (1.281, 2.418)	<0,001	1.909 (1.392, 2.617)	<0,001
Psoriasis	6496	120	1.661 (1.241, 2.224)	0,001	1.794 (1.342, 2.396)	<0,001
Sarcoidosis	6678	15	1.502 (0.674, 3.345)	0,316	2.055 (0.871, 4.846)	0,093
Sjörgen syndrome	6627	70	1.643 (1.122, 2.406)	0,010	1.796 (1.228, 2.626)	0,002
Systemic involvement of connective tissue, unspecified	6560	80	4.250 (2.574, 7.017)	<0,001	4.614 (2.798, 7.609)	<0,001
Systemic lupus erythematosus	6639	46	3.319 (1.823, 6.043)	<0,001	3.591 (1.974, 6.533)	<0,001
Thyrotoxicosis with diffuse goiter	6676	17	1.420 (0.677, 2.975)	0,351	1.554 (0.742, 3.255)	0,238
Type 1 diabetes mellitus	6540	64	1.135 (0.793, 1.625)	0,488	1.236 (0.865, 1.767)	0,243
Ulcerative colitis	6641	35	0.816 (0.522, 1.277)	0,373	0.898 (0.575, 1.404)	0,637
Vitiligo	6678	≤ 10 *	-- **	--	1.980 (0.663, 5.908)	0,212
* Counts of less than and equal to 10 will show as ≤ 10						
** The odds ratio isn't sufficiently reliable due to the limited number of patients.						

Supplementary Table 5 : Black/African American Group Analysis						
**Outcome Name**	**Patients in cohort**	**Patients with outcome**	**OR (95% CI)**	**P-value**	**HR (95% CI)**	**Log- Rank P-value**
Alopecia areata	1066	≤ 10 *	-- **	--	2.238 (0.409, 12.239)	0,34
Antiphospholipid syndrome	1068	≤ 10 *	--	--	--	0,08
Autoimmune thyroiditis	1071	≤ 10 *	-- **	--	1.134 (0.159, 8.083)	0,90
Behçet’s disease	1068	0	-- **	--	--	1,00
Bullous pemphigoid	908	82	--	<0,001	--	<0,001
Celiac disease	1072	≤ 10 *	-- **	--	0.738 (0.123, 4.415)	0,74
Cicatricial pemphigoid	1045	13	--	<0,001	--	<0,001
Crohn’s disease [regional enteritis]	1068	≤ 10 *	-- **	--	1.107 (0.156, 7.870)	0,92
Discoid lupus erythematosus	1055	≤ 10 *	-- **	--	10.176 (1.288, 80.399)	0,01
Eosinophilic esophagitis	1072	≤ 10 *	-- **	--	1.595 (0.266, 9.549)	0,61
Hemolytic anemias	1053	12	0.630 (0.304, 1.305)	0,21	0.722 (0.350, 1.490)	0,38
Immune thrombocytopenic purpura	1069	≤ 10 *	-- **	--	0.539 (0.049, 5.946)	0,61
Lichen planus	1057	12	1.217 (0.524, 2.830)	0,65	12.723 (1.654, 97.858)	<0,001
Multiple sclerosis	1066	0	-- **	--	--	0,18
Myasthenia gravis	1070	≤ 10 *	-- **	--	--	0,29
Neutropenia	1058	13	0.936 (0.438, 2.000)	0,86	1.030 (0.484, 2.192)	0,94
Other rheumatoid arthritis	1024	20	2.076 (0.967, 4.456)	0,06	2.595 (1.181, 5.702)	0,01
Psoriasis	1039	17	1.760 (0.802, 3.862)	0,15	3.864 (1.424, 10.480)	<0,001
Sarcoidosis	1063	≤ 10 *	-- **	--	1.107 (0.277, 4.430)	0,89
Sjörgen syndrome	1063	11	1.107 (0.468, 2.618)	0,82	2.060 (0.761, 5.574)	0,15
Systemic involvement of connective tissue, unspecified	1112	16	1.609 (0.727, 3.561)	0,24	8.415 (1.935, 36.604)	<0,001
Systemic lupus erythematosus	1046	≤ 10 *	-- **	--	--	<0,001
Thyrotoxicosis with diffuse goiter	1062	≤ 10 *	-- **	--	9.740 (1.234, 76.885)	0,01
Type 1 diabetes mellitus	1042	23	1.457 (0.765, 2.774)	0,25	1.583 (0.836, 2.997)	0,16
Ulcerative colitis	1070	≤ 10 *	-- **	--	3.028 (0.803, 11.422)	0,09
Vitiligo	1066	≤ 10 *	-- **	--	3.344 (0.674, 16.588)	0,12
* Counts of less than and equal to 10 will show as ≤ 10						
** The odds ratio isn't sufficiently reliable due to the limited number of patients.						

Supplementary Table 6 : Asian Group Analysis						
**Outcome Name**	**Patients in cohort**	**Patients with outcome**	**OR (95% CI)**	**P-value**	**HR (95% CI)**	**Log- Rank P-value**
Alopecia areata	1038	≤ 10 *	-- **	--	0.691 (0.115, 4.140)	0,684
Antiphospholipid syndrome	1034	15	1.513 (0.677, 3.384)	0,31	16.869 (2.226, 127, 819)	<0,001
Autoimmune thyroiditis	1016	15	1.395 (0.638, 3.052)	0,403	1.467 (0.673, 3.195)	0,332
Behçet's disease	947	≤ 10 *	-- **	--	--	0,005
Bullous pemphigoid	954	43	--	<0,001	--	<0,001
Celiac disease	1037	≤ 10 *	-- **	--	0.513 (0.047, 5.661)	0,579
Cicatricial pemphigoid	1014	≤ 10 *	--	--	--	0,004
Crohn's disease [regional enteritis]	1030	≤ 10 *	-- **	--	1.610 (0.268, 9.655)	0,559
Discoid lupus erythematosus	1026	≤ 10 *	--	--	--	0,003
Eosinophilic esophagitis	1037	≤ 10 *	-- **	--	0.518 (0.047, 5.708)	0,584
Hemolytic anemias	1031	≤ 10 *	-- **	--	3.204 (0.867, 11.849)	0,716
Immune thrombocytopenic purpura	1036	≤ 10 *	-- **	--	1.410 (0.316, 6.305)	0,651
Lichen planus	1008	16	1.655 (0.747, 3.664)	0,21	8.466 (1.946, 36.826)	0,001
Multiple sclerosis	1035	≤ 10 *	-- **	--	1.067 (0.150, 7.582)	0,948
Myasthenia gravis	1037	≤ 10 *	--	--	--	0,04
Neutropenia	1028	13	1.308 (0.571, 2.996)	0,525	1.933 (0.771, 4.846)	0,152
Other rheumatoid arthritis	991	21	1.570 (0.794, 3.104)	0,192	1.704 (0.866, 3.356)	0,119
Psoriasis	962	41	4.527 (2.255, 9.090)	<0,001	6.596 (2.959, 14.705)	<0,001
Sarcoidosis	1035	0	--	--	--	1,00
Sjörgen syndrome	843	57	5.690 (3.093, 10.467)	<0,001	6.187 (3.379, 11.328)	<0,001
Systemic involvement of connective tissue, unspecified	1091	46	4.758 (2.389, 9.479)	<0,001	6.884 (3.108, 15.250)	<0,001
Systemic lupus erythematosus	963	36	3.977 (1.962, 8.058)	<0,001	20.460 (4.924, 85.015)	<0,001
Thyrotoxicosis with diffuse goiter	1031	≤ 10 *	-- **	--	3.074 (0.620, 15.232)	0,147
Type 1 diabetes mellitus	1029	≤ 10 *	-- **	--	0.655 (0.214, 2.003)	0,455
Ulcerative colitis	1030	≤ 10 *	-- **	--	2.514 (0.648, 9.749)	0,167
Vitiligo	1036	≤ 10 *	--	--	--	0,08
* Counts of less than and equal to 10 will show as ≤ 10						
** The odds ratio isn't sufficiently reliable due to the limited number of patients.						

Supplementary Table 7 : Hispanic Group Analysis						
**Outcome Name**	**Patients in cohort**	**Patients with outcome**	**OR (95% CI)**	**P-value**	**HR (95% CI)**	**Log- Rank P-value**
Alopecia areata	1249	≤ 10 *	--	--	--	0,013
Antiphospholipid syndrome	1250	≤ 10 *	-- **	--	0.715 (0.119, 4.278)	0,711
Autoimmune thyroiditis	1246	≤ 10 *	-- **	--	1.501 (0.476, 4.731)	0,485
Behçet's disease	1247	≤ 10 *	-- **	--	1.038 (0.065, 16.596)	0,979
Bullous pemphigoid	1155	66	--	<0,001	--	<0,001
Celiac disease	1252	≤ 10 *	-- **	--	2.148 (0.195, 23.686)	0,523
Cicatricial pemphigoid	1228	18	--	<0,001	--	<0,001
Crohn's disease [regional enteritis]	1248	0	-- **	--	--	1,00
Discoid lupus erythematosus	1244	12	1.208 (0.520, 2.806)	0,66	6.418 (1.436, 28.678)	0,005
Eosinophilic esophagitis	1252	≤ 10 *	-- **	--	--	0,155
Hemolytic anemias	1246	0	-- **	--	--	0,172
Immune thrombocytopenic purpura	1252	≤ 10 *	-- **	--	3.651 (0.375, 35.532)	0,233
Lichen planus	1242	14	1.415 (0.626, 3.197)	0,402	16.083 (2.106, 122.802)	<0,001
Multiple sclerosis	1249	≤ 10 *	-- **	--	0.701 (0.117, 4.199)	0,696
Myasthenia gravis	1247	0	-- **	--	--	1,00
Neutropenia	1238	15	1.515 (0.678, 3.385)	0,308	2.145 (0.906, 5.079)	0,076
Other rheumatoid arthritis	1216	30	3.101 (1.509, 6.372)	0,001	7.065 (2.733, 18.261)	<0,001
Psoriasis	1226	18	1.525 (0.731, 3.179)	0,257	1.670 (0.803, 3.471)	0,165
Sarcoidosis	1251	0	-- **	--	--	1,00
Sjörgen syndrome	1237	13	1.313 (0.573, 3.005)	0,518	4.838 (1.377, 16.999)	0,007
Systemic involvement of connective tissue, unspecified	1036	16	1.609 (0.727, 3.563)	0,236	17.64 (2.338, 133.070)	<0,001
Systemic lupus erythematosus	1236	≤ 10 *	-- **	--	1.065 (0.308, 3.679)	0,921
Thyrotoxicosis with diffuse goiter	1246	≤ 10 *	-- **	--	0.410 (0.042, 4.016)	0,429
Type 1 diabetes mellitus	1230	20	1.833 (0.875, 3.842)	0,103	1.993 (0.954, 4.163)	0,061
Ulcerative colitis	1250	≤ 10 *	-- **	--	1.786 (0.427, 7.475)	0,421
Vitiligo	1248	≤ 10 *	-- **	--	3.805 (0.790, 18.331)	0,073
* Counts of less than and equal to 10 will show as ≤ 10						
** The odds ratio isn't sufficiently reliable due to the limited number of patients.						
